# Has the Flood Entered the Basement? A Systematic Literature Review about Machine Learning in Laboratory Medicine

**DOI:** 10.3390/diagnostics11020372

**Published:** 2021-02-22

**Authors:** Luca Ronzio, Federico Cabitza, Alessandro Barbaro, Giuseppe Banfi

**Affiliations:** 1 Department of Informatics, University of Milano-Bicocca, 20126 Milan, Italy; l.ronzio@campus.unimib.it; 2 IRCCS Istituto Ortopedico Galeazzi, Via Riccardo Galeazzi, 4, 20161 Milan, Italy; barbaro.alessandro.ab@gmail.com (A.B.); banfi.giuseppe@fondazionesanraffaele.it (G.B.); 3 School of Medicine, University Vita-Salute San Raffaele, Via Olgettina, 58, 20132 Milan, Italy

**Keywords:** laboratory medicine, machine learning, deep learning, laboratory tests

## Abstract

This article presents a systematic literature review that expands and updates a previous review on the application of machine learning to laboratory medicine. We used Scopus and PubMed to collect, select and analyse the papers published from 2017 to the present in order to highlight the main studies that have applied machine learning techniques to haematochemical parameters and to review their diagnostic and prognostic performance. In doing so, we aim to address the question we asked three years ago about the potential of these techniques in laboratory medicine and the need to leverage a tool that was still under-utilised at that time.

## 1. Introduction

In 2018, we published a survey of the existing literature on the use of machine learning (ML) techniques in laboratory medicine, as reported in studies published from 2007 to 2017 [1]. In that effort, we noticed that very few works reported on the use of ML techniques to address either diagnostic or prognostic tasks, leveraging a large amount data extracted from laboratory tests, especially in comparison to the number of studies reporting on the application of ML techniques to diagnostic imaging and instrumental examinations. We summarised this seeming paradox, claiming that the “flood” of works applying ML to laboratory medicine had not yet occurred. Three years later, this current review aims to obtain an up-to-date picture of the body of work reporting on the use of ML applications in laboratory medicine that were published from 2018 to 2020.

After our first review, in 2019, Naugler and Church [2] discussed the innovative potential of the current techniques of artificial intelligence (AI) in laboratory medicine, and they highlighted the significant amount of attention being paid to and the increased expectations associated with cognitive computing from multiple stakeholders and standpoints in the medical community. That interest has constantly increased since then: in 2019, a MEDLINE search using the expression “artificial intelligence” as the query would approximately return 25,000 papers; in contrast, in late 2020, the same query yielded almost 110,000 articles, more than a four-fold increase within one year [3].

The interest in ML is not confined to the academic world, given the increasing number of AI-based algorithms approved by the United States Food and Drug Administration (FDA) [4]. Out of 68 models approved by the FDA, 61 were cleared during the last three years, especially for radiology applications, with 34 algorithms that can be traced back to this medical field. Cardiology, with 18 models, is another area where some form of AI technology is proposed more frequently [5].

Conversely, laboratory medicine is not yet very well represented in this context. Only seven models have been registered to date: one of these is an application to detect urinary tract infections [6] and another is a system capable of predicting blood glucose changes over time [7].

In this article, we address the academic production and reporting of ML studies as a natural follow-up of our first review. While in our first review we provided an introduction to the ML approach for a medical readership, here, we only mention the main elements and definitions (interested readers can refer to Reference [1] or [8,9,10] for more details).

ML is an umbrella term for a number of computational and statistical techniques that can be used to create a medical AI, which is a system that is capable of automating, or computationally supporting, a complex medical task, such as diagnosis, prognosis or treatment planning and monitoring [1,10,11]. Specific definitions of ML usually vary according to the field and area of interest of their source. The definition of ML that we want to address in this paper is a combination of two recent contributions by Wang, Summers and Obermeyer [12,13]: ML is “the study of how computer algorithms can ‘learn’ complex relationships or patterns from empirical data and produce (mathematical) models linking a large number of covariates to some target variable of interest” [8]. Although this definition mentions the common term, ‘learn’, its meaning should be considered as an evocative way to denote the iterative optimisation of a mathematical model (or function), rather than something related to the human ability to learn and acquire new knowledge and skills [10]. The field of computer science is full of metaphorical expressions that refer to human capabilities that do not have a real counterpart in the computational field [9], and ML is no exception, as it includes the term deep learning (DL), which denotes a subset of ML techniques that employ a class of mathematical models known as artificial neural networks (ANNs). While DL models exhibit a generally high accuracy in diagnostic tasks based on digital imaging (such as Computed tomography (CT), X-rays and Magnetic resonance imaging (MRI)), their deepness refers to the multi-layered nature of the computational models and not some deep comprehension of the input received, or any insight achieved. In fact, for tabular and numerical data, such as data obtained from laboratory exams, other ML methods, including logistic regression, have been found to be preferrable [14], especially in terms of their higher generalisability and interpretability [15].

The three main tasks executed by ML models are classification, regression and clustering. Classification, as the word infers, refers to the identification, for each record given to the model as input, of its target class or correct category. Regression refers to the estimation of the correct value of a continuous variable. Clustering is an approach that allows one to group different records together by associating them to groups (i.e., clusters) on the basis of their similarities.

## 2. Materials and Methods

**Figure 1:**
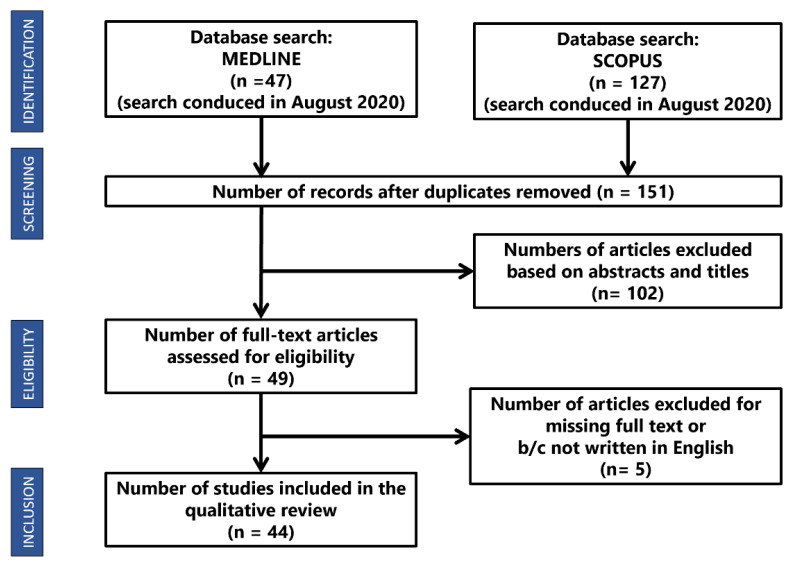
Description of the sample of surveyed articles.

In August 2020, we performed the searches reported in Figure 1 on PubMed Central^®^ (PMC), which is a free archive of biomedical and life sciences journal literature managed by the United States National Institutes of Health’s National Library of Medicine, and Scopus, a comprehensive abstract and citation database curated by Elsevier. The query we chose is reported in Appendix A.

Our objective was to find the published articles in which their authors claimed to have used ML for a task connected to laboratory medicine. A first selection of papers was obtained by searching for specific keywords in the titles and abstracts only, and, when deemed necessary, on the content of the whole manuscript. With respect to the indications provided by Salvador-Olivan [16], the search query included the base form of the words, and particular attention was paid to the use of Boolean operators or parenthesis. Our inclusion criteria were:

- The study was written in English.
- The study was published after March 2017.
- The study mentioned at least one known ML technique.
- The study was available online as a full research article or review.

Studies that did not meet all these criteria were excluded. Then, we performed a qualitative analysis of the papers that were obtained from this first selection process. Subthemes for each article were defined and relevant labels were assigned following the Grounded Theory approach [17]. The subthemes were then organised into main themes, in order to avoid overlap and redundancy. If a new subtheme emerged, all the papers were re-evaluated to apply the new subtheme in an iterative way, known as the constant comparison method [17].

For each paper, we extracted and summarised the following information: the medical domain the study belonged to, the kind of cohort involved, the purpose of the application, the ML technique applied and the evaluation metrics used to assess the model’s performance.

## 3. Results

Our research produced 127 results from Scopus and 47 from PubMed. Among these, 23 were duplicates, so a total of 151 unique articles were retrieved. After reviewing the titles and abstracts, 102 papers were excluded. Four more papers were ignored because they were not written in English and one was rejected because the full text was not available. Finally, 44 articles were analysed, and the main characteristics of each are reported in Table 1 and Table 2.

All the papers included in the review were published from 2017 to 2020, as shown in Table 1 and Table 2. Notably, almost three-quarters of these studies (n = 31, 71%) were published in the last two years.

**Figure 2:**
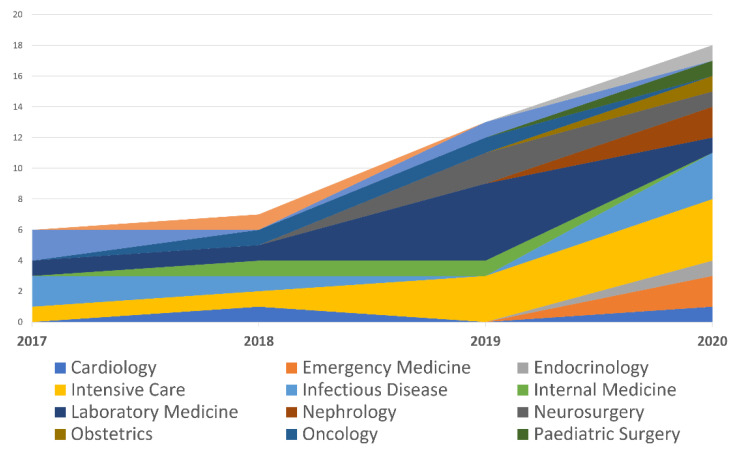
Published papers based on their medical specialty.

From a study design standpoint, virtually all of the papers used a retrospective approach (96%). Table 3 and Figure 2. depict the number of articles divided by medical specialty in association with laboratory medicine.

The prognostic task was the one that was most often represented (48%), followed by the diagnostic task (30%): 18% of the articles had a research task and 4.6% had a therapy task. When included, detection (64%) was the type of analysis most often conducted, followed by regression (16%) and characterisation (9.1%).

**Figure 3:**
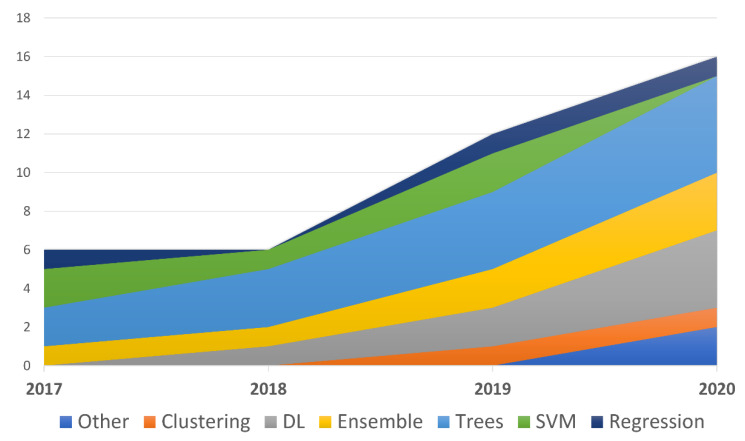
The families of the best performing models described in the articles based on the year of publication.

Most of the studies proposed multiple comparisons between several ML models, while only eight proposed a simpler comparison between two models. The models that were tested more frequently are (in decreasing order): models based on decision trees (DTs), such as random forest (RF), regression models, Ensemble models, support vector machines (SVMs) and DL. If we only consider the best performing models, or those specifically recommended by the authors, the trees family of models (especially RF and DT), Ensembles (e.g., XGBoost) and DL models represent the majority, as shown in Figure 3.

Given the non-homogeneity of the model tasks, many metrics were used. Of those, area under the receiver operating curve (AUROC), sensitivity and specificity, F-score, positive predictive value (PPV) and negative predictive value (NPV) were the metrics most frequently adopted and reported.

As this is a systematic review that does not address a single clinical problem, we decided to not describe the articles by grouping them within macro-arguments, as other authors did in their narrative reviews (see References [62,63]). Instead, we will report one single model for each medical specialty, chosen on the basis of its potential clinical implications from the most recent and the most relevant studies considered in our survey.

### 3.1. Cardiology: Machine Learning Can Predict the Survival of Patients with Heart Failure from Serum Creatinine and Ejection Fraction Alone

Heart failure and the best features for predicting the outcome of patients suffering from this disease were the topics of the article from Chicco and Jurman [50]. They studied 299 patient records containing 13 features (clinical, body and lifestyle information) to predict the death or survival rates within 130 days. Ten different ML models—linear regression, RF, one rule, DT, ANN, two SVMs, k-nearest neighbours (KNN), naïve Bayes (NB) and gradient boosting (GB)—were trained and validated on the respective sets using all 13 predictors. On the testing set, RF outperformed all the other methods in terms of the Matthew correlation coefficient (MCC), which was the most relevant for the authors, obtaining an MCC of +0.384, and in terms of accuracy (0.740) and AUROC (0.800). Subsequently, from a series of univariate analysis and Gini impurity (from RF), the authors determined that serum creatinine and ejection fraction were the most important features for the outcome prediction for all the methods. They again trained three ML algorithms (RF, GB, SVM radial) using only these two features and obtained a better result than they achieved the first time. The second time, the best performer (again RF) obtained an MCC of +0.418, and an accuracy of 0.585, but a lower AUROC than GB (0.698 vs 0.792). Finally, the authors introduced the follow-up period (in months) as a temporal variable in a stratified logistic regression model, which obtained a better result than the model that only used creatinine and ejection fraction. Moreover, surprisingly, that model outperformed all the other models (MCC: +0.616; accuracy: 0.838; AUROC: 0.822; True Positive Rate (TPR): 0.785; True Negative Rate (TNR): 0.860).

### 3.2. Emergency Medicine: Predicting Adverse Outcomes for Febrile Patients in the Emergency Department Using Sparse Laboratory Data: Development of a Time-Adaptive Model

Timeliness is crucial, especially in the context of an emergency department. In Reference [47], Lee et al. developed time adaptive models that predict adverse outcomes for febrile patients (T > 38°) using not only the values of lab tests (order status and results (OSR)), but also the simple request (order status only (OSO)). For this purpose, five ML algorithms (RF, SVM, logistic regression, ridge and elastic net regularization) were trained and validated using 9491 patients and variables chosen by experts. Of these, RF was the best performing ML model: it obtained an Area Under the Curve (AUC) of 0.80 (0.76–0.84) for OSO and 0.88 (0.85–0.91) for OSR. Comparing it with Modified Early Warning Score (MEWS), a reference algorithm used to predict the severity of a patient, the RF ML model showed an AUC improvement of 12% for OSO, meaning that the order pattern can be valuable in terms of predictions with a consistent saving time, and an AUC improvement of 20% for OSR. The RF and elastic net OSO models had Troponin I, creatine kinase and Creatine kinase isoenzyme MB (CK-MB) as the three top variables, while the lactic acid test was the most important variable for OSR.

### 3.3. Endocrinology: Identification of the Risk Factors for Patients with Diabetes: Diabetic Polyneuropathy Case Study

In Reference [60], Metsker et al. tackled the problem of predicting the risk of polyneuropathy in diabetic patients. To find the best way to handle missing data, they chose different solutions and obtained six different datasets. A T-distributed stochastic neighbour embedding (T-SNE) algorithm was applied to those datasets and data from 5425 patients were clustered in six subclasses. Five ML models (ANN, SVM, DT, linear regression and logistic regression) were trained first using 29 features and then 31 features (comorbidities were added to the previous 29). The performance on the testing set differed depending on whether the model was trained on 29 or 31 variables. Among the models with 29 variables, the best accuracy (0.7472) and best F1 score (0.7299) were obtained using the linear regression model. The logistic regression model had the best precision (0.6826), and ANN had the best sensitivity (0.8090). Among the models trained on all the variables, which obtained generally better results, ANN outperformed all the other models for every evaluation metric (sensitivity: 0.8152, F1 score 0.8064, accuracy: 0.8261, AUC: 0.8988) except for precision, in which SVM performed the best (0.8328). From the correlation analysis, it was then confirmed that both the patient’s age and mean platelet volume have a positive correlation with polyneuropathy. Moreover, the DT model showed that the development of polyneuropathy is associated with the reduction in the relative number of neutrophils.

### 3.4. Intensive Care: Early Diagnosis of Bloodstream Infections in the Intensive Care Unit Using Machine Learning Algorithms

Roimi et al. [54] developed an ML model that can predict intensive care unit-acquired bloodstream infections (BSIs) among patients suspected of infection. They conducted a bi-centre study using data from the Beth Israel Deaconess Medical Center (BIDCM) database system (MIMIC-III) and data from the Rambam Healthcare Campus (RHCC) database system. Respectively, 2351 and 1021 patient records were included in the analysis and many features (demographics, clinical, lab tests, medical treatment and time-series variables, generated after collection) were considered, although not all the features were included in both databases. To avoid overfitting, an XGBoosting feature selection algorithm was applied to the two datasets. On each dataset (BIDCM and RHCC), previously split into a training subset and a validation subset, a version of the model was trained. The model was an ensemble of six RF models and two XGBoost models, tuned with different settings. The final result was provided by the soft voting method applied to the probability of the BSI outputted by each single algorithm. After conducting 10-fold cross-validation, the models were tested: the BIDCM model obtained an AUROC of 0.89 ± 0.01 and a Brier score of 0.037 (0.90 ± 0.01 and 0.047 considering a variation of the dataset), and the RHCC model obtained an AUROC of 0.92 ± 0.02 and 0.098 (0.93 ± 0.03 and 0.061), respectively. In comparison to the single ML models (LASSO, RF and GB tree (GBT)), the proposed model outperformed all the others. Finally, the authors performed an external validation, running one model on data from the other’s database. They found that the AUROCs deteriorated: the AUROC was 0.59 ± 0.07 for BIDMC and 0.60 ± 0.06 for RHCC. Although many variables were different between the databases, that was not the reason for the loss of performance.

### 3.5. Infectious Disease: Routine Laboratory Blood Tests Predict SARS-CoV-2 Infection Using Machine Learning

The Sars-CoV-2 pandemic and the global unpreparedness to address it was an important stimulus for identifying a new way to face the associated problems; for example, the difficulty of having specific tests drove the medical community to look for new approaches. Yang et al. [44] evaluated 3356 eligible patients. They constructed a 685-dimensional vector made of laboratory tests results for each patient, reducing it to a 33-dimension vector (27 lab analysis, age, gender and ethnicity), according to the results of the univariate analysis that was performed to assess the association with the real-time reverse transcriptase polymerase chain reaction (RT-PCR) result (considered ground truth for positiveness to SARS-CoV-19). Four ML models were trained considering these features (logistic regression, DT, RF, gradient boosting decision tree (GBDT)), and they were evaluated in two different settings. The best performance was obtained by GBDT (AUC = 0.854, 95% confidence interval (CI) 0.829–0.878) in the first setting, while in the second setting, the AUC was 0.838. Using the Shapley additive explanations technique, the authors found that the most important variables for the model were lactic acid dehydrogenase (LDH), C-reactive protein (CRP) and ferritin.

### 3.6. Internal Medicine: A Real-Time Early Warning System for Monitoring Inpatient Mortality Risk: Prospective Study Using Electronic Medical Record Data

Ye et al. [43] developed and validated a real-time early warning system (EWS) designed to predict patients at high risk of mortality in order to assist clinical decision making and to enable clinicians to focus on high-risk patients before the acute event. First, the observation window, called “inpatient day”, was set (24 h), and 680 predictors were chosen among the historical medical variables and clinical information. Two different cohorts were involved in the study. The first was a retrospective cohort consisting of 42,484 patients, and it was used to build the models and compare them. RF, XGBoost, Boosting, SVM, LASSO and KNN were the models chosen to predict the outcomes, and their PPVs were used to calculate the risk score of mortality and to determine the thresholds of risk. The second cohort, a prospective one consisting of 11,762 patients, was involved to prospectively evaluate the EWS. RF was the selected method because it obtained the highest c-statistic (0.884). A risk score was assigned to each observation window (inpatient day), and then these were stratified. Considering high-risk patients, EWS achieved a sensitivity of 26.7% (68/255 death patients) and a PPV of 69%, successfully alerting clinicians from 24–48 h to 7 days before the death of 68 out of 99 of the high-risk patients. Comparing EWS with VitalPAC early warning score (ViEWS), a common warning score, this latter was outperformed, since it showed a c-statistic of 0.764, sensitivity of 13.7 and a PPV of 35%. Considering both high- and intermediate-risk patients, the new EWS was better. Finally, applying the Gini index, 349 predictors strongly associated with the outcome were recognised, and they included the expected cardiovascular disease, congestive heart failure or renal disease, but curiously, also emergency department visits, inpatient admissions and the clinical costs incurred over the previous 12 months.

### 3.7. Laboratory Medicine: Predict or Draw Blood: An Integrated Method to Reduce Lab Tests

A stimulating field of investigation is represented by the ability of ML models to predict laboratory test values without performing them. Yu et al. [49] developed a neural network model with the aim of reducing the number of tests performed, losing only a small percentage of accuracy. Using data from 12 lab tests obtained from the MIMIC-III dataset, they trained their models, which consisted of two modules. The first module predicted the laboratory result, while the second predicted the probability, according to a threshold, that the test would be conducted. The best model was the one that considered not only the lab test but also the demographic, vital signs and an encoding indicating missing values. Given the definition of “accuracy” as the proportion of concordant pairs between the predicted state and the observed state (normal/abnormal), it was found that the model using a 33% reduction in the number of tests was able to maintain an accuracy of more than 90%, while the model using a 15% reduction was able to maintain an accuracy of more than 95%.

### 3.8. Nephrology: A Recurrent Neural Network Approach to Predicting Haemoglobin Trajectories in Patients with End-stage Renal Disease

Many patients that required haemodialysis and received erythropoiesis-stimulating agents (ESA) due to end-stage renal diseases, experience the haemoglobin (Hgb) cycling phenomenon. Data from 1972 patients allowed Lobo et al. [53] to develop a recurrent neural network (RNN) that used historic data, future ESA and iron dosing data to predict the trajectory and the Hgb levels within the following 3 months. Among the patient characteristics, dialysis data, dosing of ESA and laboratory tests (haemoglobin and iron included), 34 variables were chosen by nephrologists and after close examination of the literature. A three-module neural network model was then built. The first module, an RNN-long short-term memory (RNN-LSTM), was used to compute the history of patients as a weekly time series. The second module, a regular neural network, elaborated the static data. The third module, another RNN-LSTM, encompassed the future weekly doses of ESA and iron over the forecasting horizon. In addition to the variables, the weekly time series for clinical events was added and seven parameters were used to make different combinations. Due to the three different forecasting horizons (1, 2 and 3 months), 960 different models were trained. As expected, the greater the number of months that were included in the history, the better the performance. Similarly, the performances were better when a near forecast was asked of the model. In contrast to commonly held beliefs, the best performances were obtained from the models that used a small number of features and a smaller version of the model. In fact, the best performance was obtained by the model that analysed 5 months of data and had its forecasting horizon set at 1 month, used less features and a simpler network. Its mean absolute error (MAE) was 0.527 and its mean squared error (MSE) was 0.489. The authors reported that running the model without the future iron dosing information allowed them to obtain comparable results. Moreover, they claimed to have provided a system that can predict the trend of haemoglobin according to the therapy in order to allow clinicians to forecast what would happen if they did or did not administer the planned therapy.

### 3.9. Neurosurgery: Feasibility of Machine Learning-based Predictive Modelling of Postoperative Hyponatremia after Pituitary Surgery

The aim of the study conducted by Voglis et al. [61] was to evaluate whether an ML model could predict postoperative hyponatremia (i.e., serum sodium < 130 mmol/L within 30 days after surgery) after resection of pituitary lesions. A total of 26 features (laboratory findings and from pre- and post-operative MRI results) from the data of 207 patients were used to train and test four different ML models: generalized linear model (GLM), GLMBoost, NB classifier and RF. For the missing data, a KNN algorithm was used. During the validation, the GLMBoost model delivered the best performance with an AUROC of 67.1, an F1 score of 40.6%, a PPV of 35.3 and an NPV of 82.5. The NB model obtained the best sensitivity (73.4 vs 47.8), and RF had the best accuracy (69.3 vs 67.7). Only GLMBoost was run on the testing dataset, showing an AUROC of 84.3% (95% CI 67.0–96.4) and an accuracy of 78.4% (95% CI 66.7–88.2). The sensitivity was 81.4%, the specificity was 77.5% and the F1 score was 62.1%. Due to the low prevalence of the condition in the patient population, this last model obtained a high NPV (93.9%) but a low PPV (50%). Assessing the loss in performance of the model when each variable was rejected, the most important features were identified: preoperative serum prolactin, preoperative serum insulin-like growth factor 1 level (IGF-1), body mass index (BMI) and preoperative serum sodium level.

### 3.10. Obstetrics: Comparison of Machine Learning Methods and Conventional Logistic Regressions for Predicting Gestational Diabetes Using Routine Clinical Data: A Retrospective Cohort Study

Ye et al. [51] compared the performance of many ML models and logistic regression for predicting gestational diabetes (GDM) using routine lab tests. They chose 104 variables (medical history, clinical assessment, ultrasonic screening data, biochemical data and data from Down’s screening), and they included 22,242 women in the study. Eight ML models (GBDT, AdaBoost, light gradient boosted (LGB), Vote, extreme gradient boosted (XGB), RF, DT, ML logistic regression) and two conventional logistic regression models were trained, tested and compared. Concerning discrimination, GDBT was the best performer among the ML models, although the logistic regression model was found to have a similar AUC (73.51%, 95% CI 71.36%–75.65% vs 70.9%, 95% CI 68.68%–73.12%). In terms of calibration, GDBT was the second-best performer after DT. According to the GBDT model, fasting blood glucose, glycated haemoglobin, triglycerides and maternal body mass index (BMI) were the most important predictors, while HDL and glycated haemoglobin were the most important, according to the logistic regression model. In the GBDT model, the authors identified 0.3 as the point to predict the absence of GDM with an NPV of 74.1 (95% CI 69.5%–78.2%) and a sensitivity of 90% (95% CI 88.0%–91.7%), and they identified 0.7 as the point to predict the presence of GDM with a PPV of 93.2% (95% CI 88.2%–96.1%) and a specificity of 99% (95% CI 98.2%–99.4%). According to the authors, the ML models did not outperform the conventional logistic regression models.

### 3.11. Oncology: Survival Outcome Prediction in Cervical Cancer: Cox Models vs. Deep-learning Model

Matsuo et al. [37] conducted a study involving 768 women with the aim of comparing the ability of the most important tool for survival analysis on oncologic research. They applied the Cox proportional-hazards (CPH) regression model, a DL model particularly suited for predicting survival in women with cervical cancer. Three groups of features were chosen (20 features about vital signs and lab tests, 16 additional features about the tumour and 4 about treatment). They trained five baseline models (CPH, CoxLasso, random survival forest and CoxBoost), and a DL model. The tasks were the prediction of progression-free survival (PFS) and the prediction of overall survival (OS), which were described using MAE and the concordance index calculated as the average of 10-fold evaluations. Using the third group of features (the largest one), the DL model outperformed the CPH model for PFS (CI 0.795 ± 0.066 vs. 0.784 ± 0.069, and MAE 29.3 ± 3.4 vs. 316.2 ± 128) and for OS (CI 0.616 ± 0.041 vs. 0.607 ± 0.039, and MAE 30.7 ± 3.6 vs. 43.6 ± 4.3). The performance of all the other models was similar to that of the DL model. Both DL and CPH were in agreement about the importance of blood urine nitrogen (BUN), albumin and creatinine for PFS prediction, BUN for OS prediction, while only the DL model used white blood cell (WBC) count, platelets, bicarbonate and haemoglobin for PFS and bicarbonate for OS, surprisingly omitting albumin, creatinine and platelets, which were used by the CPH model. Given the omission of albumin, a well-recognised prognostic factor, the authors expressed their concern about the reliability of the model.

### 3.12. Paediatric Surgery: A Novel and Simple Machine Learning Algorithm for Preoperative Diagnosis of Acute Appendicitis in Children

Aydin et al. [59] considered data from 7244 patients to develop a simple algorithm for preoperative diagnosis of appendicitis in children. They trained six ML models (NB, KNN, SVM, DT, RF and generalised linear model) and tested them. Although DT was not the best performer (the AUC was 93.97 for DT vs 99.67 for RF), it was the model they were looking for because it was simple, easy to interpret and familiar to clinicians. It also provided a clear interpretation of the importance of the variables: platelet distribution width (PDW), WBC count, neutrophils and lymphocytes were the most important factors for detecting appendicitis in patients. In the analysis to assess whether the DT model was able to differentiate patients with complicated appendicitis, its performance further decreased (AUC of 79.47%, accuracy of 70.83, sensitivity of 66.81%, specificity of 81.88%).

### 3.13. Paediatrics: Enhanced Early Prediction of Clinically Relevant Neonatal Hyperbilirubinemia with Machine Learning

The goal of the study conducted by Daunhawer et al. [39] was to predict, after each bilirubin measurement, if a neonate would develop an excessive bilirubin level in the next 48 h. Toward that end, 44 variables from 362 neonates were used to assess three different models: a LASSO model (L-1 regularized logistic regression), an RF model and a model that combined the predictions of the previous two. The combined model had an AUC of 0.592 ± 0.013, while the LASSO and RF models had an AUC of 0.947 ± 0.015 and 0.933 ± 0.019, respectively. RF, a backward selection, and LASSO were also used to identify gestational age, weight, bilirubin level and hours since birth, and they were found to suffice for a strong predictive performance, as the most strongly associated variables. The authors developed an online tool using the best performing model, and they validated it on an external dataset, thus obtaining a better AUROC (0.954).

### 3.14. Pharmacology: Machine Learning Model Combining Features from Algorithms with Different Analytical Methodologies to Detect Laboratory-event-related Adverse Drug Reaction Signals

In Jeong et al. [25], the problem of identifying and evaluating adverse drug reactions was addressed using a ML model that integrates already existing algorithms based on electronic health record (EHR). From the Ajou University Hospital EHR dataset, the European Union Adverse Drug Reactions from Summary of Product Characteristics (EU-SPC database) and Side Effect Resource (SIDER) 4.1, a resource of side effects extracted from drug labels, they made an adverse drug reaction (ADR) reference dataset of 1674 drug–event pairs (778 with known associations and 896 with unknown associations). The outputs and intermediates of Comparison of Extreme Laboratory Test (CERT), Comparison of Extreme Abnormality Ratio (CLEAR) and Prescription pattern Around Clinical Event (PACE) algorithms (18, 25 and 5, respectively) were extracted and used as features for four ML models, more precisely, L1 regularized logistic regression, RF, SVMs and NNs. The performances of older algorithms (i.e., CLEAR, CERT400, CCP2) were then compared to the average performance of each ML model, which were evaluated based on 10 experiments with a 10-fold cross-validation for each model. The ML algorithm outperformed the other algorithms. The F-scores and the AUROCs of the ML models were 0.629–0.709 and 0.737–0.816 respectively, instead of 0.020–0.597 and 0.475–0.563 respectively, from the older methods. RF had the highest AUROC and PPV (0.727 ± 0.031), while NN the highest sensitivity (0.793 ± 0.062), NPV (0.777 ± 0.052) and F-scores (0.709 ± 0.037). SVM had the highest specificity (0.796 ± 0.046). By using the Gini index in the RF model and the magnitude of coefficient in the L1 regularized logistic regression model, they found that the most important features were related to the shape of the distribution and the descriptive statistics of laboratory result tests.

### 3.15. Urology: Dynamic Readmission Prediction using Routine Postoperative Laboratory Results after Radical Cystectomy

Kirk et al. [55] used data from 996 patients to assess if the integration of routine postoperative data in a predictor model of 30-day readmission after cystectomy could improve its predictive performance. Demographic, laboratory-related and complication-related variables were considered, and a SVM model was used to define the daily (1 to 7 days after discharge) cut-offs to distinguish between readmitted and non-readmitted patients. Multiple logistic regression models were trained using different combinations of variables and thresholds from SVM, and clinical data were used to examine the effects on readmission risk. The most discriminative values were WBC, bicarbonate, BUN and creatinine, whereas BUN, WBC, total bilirubin and chloride showed greater variance in the readmitted patients than in the non-readmitted ones. Among the models, the best performance was obtained from the one that included all the variables, the SVM thresholds and postoperative complications (AUC = 0.62); however, by adding lab test thresholds, it was possible to improve the performance (AUC of 0.59 for the SVM model vs AUC of 0.52 for the previous model). Finally, using the same variables that were used in the best-performing model, the authors also trained an RF regression model that achieved an AUC of 0.68.

## 4. Discussion

In the previous literature review [1], 37 papers (published between 2007 and 2017) were found, of which only three were indexed by the MEDLINE database. In the review presented here, we found 44 articles, with a significant yearly increase: from six articles in 2017 to 18 in the first 8 months of 2020. Although this can be interpreted as a sign of growing interest, it should be noted that almost all of the articles were retrospective studies, with two exceptions [43,56].

As seen in the previous section, the models based on decision trees were the most popular. In particular, the RF model was selected by several authors [18,22,29,34,38,42,43,47,48,50,55], while DTs were only chosen in two relevant studies [24,59]. This could be due to a variety of reasons, such as the generally very good performance of this class of models, and because of their interpretable output, especially when this is enriched with estimates of the most relevant variables expressed in terms of the Gini impurity index. However, clarity and simplicity are the reasons why logistic regression was also frequently chosen [36,56], both as a stand-alone model and also as a baseline model to be compared with other, more complex (and hence less generalisable) models. Among the best models, those in the Ensemble family (e.g., XGB, GBT) were chosen both for their medium–high performance [21,30,32,33,44,51,58,61] and their training speed. Models in the DL family [27,35,37,49,52,53,60], especially RNN and ANN, have been increasingly chosen in recent years. The advantage of these systems is their potential in terms of performance, although the resources (time and the amount of data) required for training are reported to be higher for DL models than traditional ML models. RNN, and in particular RNN-LSTM, was used by several authors [27,52,53] to integrate the temporal patterns between the variables.

We can observe an evolution in the chronological accumulation of the studies considered in this review. In fact, there has been an increasing use of comparative analysis between different models with the aim of identifying the best performing model, whenever possible. This practice was not often observed before 2017, and it was seen less frequently in the first years covered by the present review than it was in 2019 and 2020, when many authors preferred to report the performance of a single algorithm, at most compared with a baseline model like logistic regression [22,26,29,39,40,41,52,56].

It is interesting to dig deeper into the purposes for which ML has been used in these studies. Although the objective of the surveyed studies was mostly "detection", that is, to answer a dichotomous question, such as whether the laboratory test is associated with either a positive or negative case or with an abnormal or normal biochemical phenotype, the authors reported a number of reasons for developing and proposing an ML approach to this class of task. One of the most frequent reasons reported was the need for systems that can predict complex conditions or outcomes more efficiently (or less costly) than is possible with longer and more expensive investigations, such as routine laboratory tests and vital parameters [23,38,54,56,58]. However, some authors [18,24,27,35,39,44] focused on the possibility of predicting or estimating the risk of certain outcomes or complications. Only rarely, and notably in Reference [43], was the impact of this anticipation evaluated, leading to interesting results, as reported in the article about internal medicine. Moreover, other authors [32,33] have dealt with how to reduce the number of analyses required to reach a conclusion through an accurate prediction of the test results, or they have assessed how to improve therapeutic protocols with complex patients [22,24,53]. Moreover, procedural problems affecting laboratory medicine, such as wrong blood in tube errors and implausible values, were analysed [26,40]. In the majority of studies, the most important variables for the models were explicitly reported. Interestingly, the variable’s importance for the model did not necessarily correlate with its biological or clinical relevance, suggesting that further research is needed to determine the potential hidden and non-trivial associations between the variables and the outcome [25,28,41].

In their conclusions, many of the authors suggested the opportunities associated with the ML approach, and some have also made their algorithms available [24,34,39,56]; however, the scarcity of articles following a prospective design shows that the interest in ML is still more academic than practical. In fact, as noted at the beginning of this article, only seven of the AI systems concerning laboratory medicine have been approved by the FDA in the last three years. This led us to believe that enthusiasm for ML is still being dampened by the need to confront the difficulties of building valid and robust models that could prove to be of use in real-world applications.

However, the lack of prospective studies can result in a lack of evaluations of the impact of ML in real-world practice; in turn, this can reduce the benefits of using ML [64]. This can occur for several reasons. Generally, it costs more to conduct prospective studies than retrospective studies. This is due to organisational reasons, data collection and cleansing, the involvement of patients and their higher risk of failure. In the context of ML, the validation on external or prospective datasets may not yield the expected results. This is true especially if the training and testing populations are different, if the model is affected by overfitting or if it has been generated in a controlled environment, hence not in real-world settings. As seen in the results, external validation is not performed regularly [65]. Even when external validation is conducted, the results are drastically inferior to the performance reported with an internal validation set [54].

However, we believe that the external validation of a model is an essential step to obtain useful tools, and it is even more necessary if the dataset is collected prospectively. Therefore, to replicate the findings reported in the studies included in this literature review, one could alternatively identify the groups of patients on whom the model is expected to work and limit its application to these subjects. A further possible reason for the relatively low number of prospective studies is the lack of bridging figures between the medical field and the information technology (IT) field. In the past 5 years, this need has led to the creation of master’s degrees that provide the basic knowledge of both biomedical engineering and medical surgery.

It follows that the choice to build an ML system is not a shortcut to obtain simpler and better results in comparison to conventional methods [51]. A good ML system requires an adequate amount of data, the right quality of the data and valid management of the missing values (i.e., data imputation), a reasoned pre-selection of the variables to input into the system and the right use of the training set, validation (or tuning) set and testing set. For instance, the articles we analysed involved a varying number of patients, ranging from populations of dozens of patients to tens of thousands. However, especially for ML systems that require large amounts of training data (e.g., DL), it is essential to utilise large amounts of complete data. Not surprisingly, intensive care and “pure” laboratory medicine were the two medical specialities that were associated with the most articles. In these two areas, it is easier to collect large amounts of data and have them available in a machine-readable format. In intensive care unit settings, this is the case because the admitted patients are usually closely monitored; in laboratory settings, this is because different machineries and modalities produce data for almost all other areas of medicine that rely on blood-related specimens.

The choice of input variables is another important aspect of a successful ML model. While DL techniques allow the use of a large number of variables because their selection and engineering are fully automated (by the input layers of ANNs), other systems are used to find the most important variables in order to focus on them [20,21,28,45,57]. Over time, what kind of variables to use is becoming clearer: it has evolved from only using static data to using simple or more complex time-related representations [27,30,48,54], which also require novel ways to manage data incompleteness and to leverage this as an indirect source of information about the patient’s condition [36]. In this regard, some authors [19,51], in light of their disappointing results, have invited the medical specialist community to choose, include and study the new predictors.

Dealing with missing data can be challenging [23]; toward that end, many different apparently effective techniques have been proposed and tested in the last 3 years [66,67,68,69]. Nevertheless, it was rare to see these techniques mentioned in the articles that were reviewed here. In 2017, it was observed that the lack of cross-citations among authors dealing with ML in orthopaedics should not be considered to be a sign of dispersion of the community of scholars who are active in ML [8]; rather, it was viewed as a sign of its heterogeneity. In light of our systematic review, we can make a similar statement by also extending it to the authors mentioned in the literature review presented in this article.

In this context, sharing the training data, even in an anonymised form, and the training details (e.g., hyperparameters, procedures of standardisation and normalisation, procedures to cope with data scarcity like k-fold cross-validation [70,71]), and hence adherence to standards for reporting ML studies properly and comprehensively (such as TRIPOD-ML [72] and CONSORT-AI [73]), is extremely important in order to enable and facilitate the reproducibility of the results and their external validation.

In spite of the partial disillusionment of some authors [65], the articles included in this review suggest that the trend toward using ML in the field of medicine will continue in the coming years. Precision (or tailored) medicine, such as the possibility to calibrate thresholds and pathological states on subjects rather than on populations [74], is a common goal of the laboratory medicine community, due to the significant amount of data available from haematochemical analysis. However, for these algorithms to be applied to daily clinical practice, we are aware that greater rigor is needed to validate clinical studies (also by applying new guidelines) and more resources are needed to create genuinely multidisciplinary research groups and to conduct more prospective studies, which could also involve more patients and a greater variety of patients.

There are a few limitations to the systematic review we conducted and reported in this article. We chose a simple but comprehensive search query, consisting of words commonly used in the area we intended to study. However, we did not use synonyms and we did not include words that are typical of subthemes or overtly technical jargon. We also only used PubMed and Scopus to conduct our search, since these are considered to be the two main academic literature indexing services.

## 5. Conclusions

Academic enthusiasm for ML in laboratory medicine is real and it is increasing. However, unlike other disciplines, laboratory medicine has not yet seemed to have embraced this perspective [5]. To determine whether the number of works applying ML to laboratory medicine has flooded the proverbial basement of this medical field, we can conclude that the flood level has certainly begun to rise, but we are still waiting for it to form a lake of consolidated knowledge and reliable tools for clinical practice.

## Figures and Tables

**Table 1:** The 44 reviewed articles showing the title, authors, year, specialty, population, features, purpose and design of the studies.

Title	Reference	Year	Specialty	Sample	Features	Design Studio	Purpose	Objective	Analysis
Early hospital mortality prediction of intensive care unit patients using an ensemble learning approach	Awad et al. (2017) [18]	2017	IC	11,722 patients (subgroups)	20–29 **	RC	Pg	To highlight the main data challenges in early mortality prediction in ICU patients and introduces a new machine learning based framework for Early (6h) Mortality Prediction for IC Unit patients (EMPICU)	De
Prediction of Recurrent Clostridium Difficile Infection (rCDI) Using Comprehensive Electronic Medical Records in an Integrated Healthcare Delivery System	Escobar et al. (2017) [19]	2017	ID	12,706	150–23 **	RC	Pg	To develop and validate rCDI predictive models based on ML in a large and representative sample of adults	De
Enhancement of hepatitis virus immunoassay outcome predictions in imbalanced routine pathology data by data balancing and feature selection before the application of support vector machines	Richardson and Lidbury (2017) [20]	2017	LM	16,990	5–27 **	RC	Dg	To use SVMs to identify predictors for the enhanced laboratory diagnosis of hepatitis virus infection, and to identify the type of data balancing and feature selection that best assisted this enhanced classification of HBV/HCV negative or positive	De
Machine Learning Algorithms for Risk Prediction of Severe Hand-Foot-Mouth Disease in Children	Zhang et al. (2017) [21]	2017	Pd	530	18	RC	Pg	To identify clinical and MRI-related predictors for the occurrence of severe HFMD in children and to assess the interaction effects between them using machine learning algorithms	De
Novel Risk Assessment Tool for Immunoglobulin Resistance in Kawasaki Disease: Application Using a Random Forest Classifier: Application Using a Random Forest Classifier	Takeuchi et al. (2017) [22]	2017	Pd	767	23	RC	Th	To develop a new risk assessment tool for IVIG resistance using RF	De
Supervised learning for infection risk inference using pathology data	Hernandez et al. (2017) [23]	2017	ID	>500,000 patients	6	RC	Dg	To evaluates the performance of different binary classifiers to detect any type of infection from a reduced set of commonly requested clinical measurements	De
Applied Informatics Decision Support Tool for Mortality Predictions in Patients with Cancer	Bertsimas et al. (2018) [24]	2018	On	23,983 patients	401	RC	Pg	To develop a predictive tool that estimates the probability of mortality for an individual patient being proposed their next treatment	Re
Machine learning model combining features from algorithms with different analytical methodologies to detect laboratory-event-related adverse drug reaction signals	Jeong et al. (2018) [25]	2018	Ph	1674 drug-laboratory event pairs	48	RC	Th	To develop a more accurate ADR signal detection algorithm for post-market surveillance using EHR data by integrating the results of existing ADR detection algorithms using ML models	De
Using Machine Learning-Based Multianalyte Delta Checks to Detect Wrong Blood in Tube Errors	Rosenbaum and Baron (2018) [26]	2018	LM	20,638 patient collections of 4855 patients	3 features for each of 11 lab tests	RC	Rch	To test whether machine learning-based multianalyte delta checks could outperform traditional single-analyte ones in identifying WBIT	De
An Interpretable ICU Mortality Prediction Model Based on Logistic Regression and Recurrent Neural Networks with LSTM units	Ge et al. (2018) [27]	2018	IC	4896	NA	RC	Pg	To develop an interpretable ICU mortality prediction model based on Logistic Regression and RNN with LSTM units	De
High-density lipoprotein cholesterol levels and pulmonary artery vasoreactivity in patients with idiopathic pulmonary arterial hypertension	Jonas et al. (2018) [28]	2018	Ca	66	NA	PC	Pg	To investigate the association between cardiometabolic risk factors and vasoreactivity of pulmonary arteries in patients with Idiopathic Pulmonary Arterial Hypertension	NE
Development and Validation of Machine Learning Models for Prediction of 1-Year Mortality Utilizing Electronic Medical Record Data Available at the End of Hospitalization in Multi-condition Patients: a Proof-of-Concept Study	Sahni et al. (2018) [29]	2018	ID	59,848	4 classes **	RC	Pg	To construct models that utilize EHR data to prognosticate 1-year mortality in a large, diverse cohort of multi-condition hospitalizations	Re
Predicting the risk of emergency admission with machine learning: Development and validation using linked electronic health records	Rahimian et al. (2018) [30]	2018	IM	4,637,297	43 + 13 **	RC	Rch	To improve discrimination and calibration for predicting the risk of emergency admission	Re
Analyte Quantity Detection from Lateral Flow Assay Using a Smartphone	Foysal et al. (2019) [31]	2019	LM	15 LFA set for 75 readings	NE	RC	Dg	To propose a robust smartphone-based analyte (albumin) detection method that estimates the amount of analyte on an LFA strip using a smartphone camera	Ch
Prevalence and Predictability of Low-Yield Inpatient Laboratory Diagnostic Tests	Xu et al. (2019) [32]	2019	LM	10,000 samples per feature	43	RC	Rch	To identify inpatient diagnostic laboratory testing with predictable results that are unlikely to yield new information	Re
Using artificial intelligence to reduce diagnostic workload without compromising detection of urinary tract infections	Burton et al. (2019) [33]	2019	LM	225,207	21	RC	Dg	To reduce the burden of culturing the large number of culture-negative samples without reducing detection of culture-positive samples	De
Interactive Machine Learning for Laboratory Data Integration	Fillmore et al. (2019) [34]	2019	LM	4 ∗ 10^9 records	NE	RC	Rch	To develop a machine learning system to predict whether a lab test type clinically belongs within the concept of interest	Ch
Early prediction of acute kidney injury following ICU admission using a multivariate panel of physiological measurements	Zimmerman et al. (2019) [35]	2019	IC	23,950	NA **	RC	Dg	To predict AKI (creatinine values in day 2 and 3) using first-day measurements of a multivariate panel of physiologic variables	De
A New Insight into Missing Data in IC Unit Patient Profiles: Observational Study	Sharafoddini et al. (2019) [36]	2019	IC	32,618–20,318–13,670 patients (days 1–2–3)	NA **	RC	Pg	To examine whether the presence or missingness of a variable itself in ICU records can convey information about the patient health status	De
Survival outcome prediction in cervical cancer: Cox models vs deep-learning model	Matsuo et al. (2019) [37]	2019	On	768	40 **	RC	Rch	To compare the deep Learning neural network model and the Cox proportional hazard regression model in the prediction of survival in women with cervical cancer	Re
Relative criticalness of common laboratory tests for critical value reporting	Yang et al. (2019) [38]	2019	IC	22,174	23	RC	Pg	To evaluate the relative strength of association between 23 most commonly ordered laboratory tests in a CCU setting and the adverse outcome, defined as death during the CCU stay within 24 h of reporting of the laboratory result	NE
Enhanced early prediction of clinically relevant neonatal hyperbilirubinemia with machine learning	Daunhawer et al. (2019) [39]	2019	Pd	362	44–4	PC	Dg	To enhance the early detection of clinically relevant hyperbilirubinemia in advance of the first phototherapy treatment	De
A clustering approach for detecting implausible observation values in electronic health records data	Estiri et al. (2019) [40]	2019	LM	>720 million records, 50 lab tests	NE	RC	Rch	To develop and test an unsupervised clustering-based anomaly/outlier detection approach for detecting implausible observations in EHR data	De
Modelling outcomes after paediatric brain injury with admission laboratory values: a machine-learning approach	Kayhanian et al. (2019) [41]	2019	Ns	94	14	RC	Pg	To identify which admission laboratory variables are correlated to outcomes after Traumatic Brain Injury (TBI) in children and to explore prediction of outcomes, using both univariate analysis and supervised learning methods	De
Automatic Machine-Learning-Based Outcome Prediction in Patients with Primary Intracerebral Haemorrhage	Wang et al. (2019) [42]	2019	Ns	1-month outcome: 307; 6-month outcome: 243	1-month outcome: 26; 6-month outcome: 22	RC	Pg	To predict the functional outcome in patients with primary intracerebral haemorrhage (ICH)	Ch
A Real-Time Early Warning System for Monitoring Inpatient Mortality Risk: Prospective Study Using Electronic Medical Record Data	Ye et al. (2019) [43]	2019	IM	42,484 retrospective, 11,762 prospective	680 **	PC	Pg	To build and prospectively validate an Early Warning System-based inpatient mortality Electronic Medical Record	Ch
Routine laboratory blood tests predict SARS-CoV-2 infection using machine learning	Yang et al. (2020) [44]	2020	ID	3356	33	RC	Dg	To develop a ML model integrating age, gender, race and routine laboratory blood tests, which are readily available with a short Turnaround Time	De
Development and validation of prognosis model of mortality risk in patients with COVID-19	Ma et al. (2020) [45]	2020	ID	305	33	RC	Pg	Investigate ML to rank clinical features, and multivariate logistic regression method to identify clinical features with statistical significance in prediction of mortality risk in patients with COVID-19 using their clinical data	De
Exploration of critical care data by using unsupervised machine learning	Hyun et al. (2020) [46]	2020	IC	1503	9	RC	Rch	To discover subgroups among ICU patients and to examine their clinical characteristics, therapeutic procedures conducted during the ICU stay, and discharge dispositions	NE
Predicting Adverse Outcomes for Febrile Patients in the Emergency Department Using Sparse Laboratory Data: Development of a Time Adaptive Model	Lee et al. (2020) [47]	2020	EM	9491	NA	RC	Pg	To develop time adaptive models that predict adverse outcomes for febrile patients assessing the utility of routine lab tests (only request OSO, and request and value OSR)	De
Temporal Pattern Detection to Predict Adverse Events in Critical Care: Case study With Acute Kidney Injury	Morid et al. (2020) [48]	2020	IC	22,542	17	RC	Pg	To evaluate approaches to predict Adverse Events in ICU settings using structural temporal pattern detection methods for both local (within each time window) and global (across time windows) trends, derived from first 48 h of ICU	NE
Predict or draw blood: An integrated method to reduce lab tests	Yu et al. (2020) [49]	2020	LM	41,113	20	RC	Rch	To propose a novel deep learning method to jointly predict future lab test events to be omitted and the values of the omitted events based on observed testing values	Re
Machine learning can predict survival of patients with heart failure from serum creatinine and ejection fraction alone	Chicco and Jurman (2020) [50]	2020	Ca	299	13–2 **	RC	Pg	To use several data mining techniques first to predict survival of the patients, and to rank the most important features included in the medical records	De
Comparison of Machine Learning Methods and Conventional Logistic Regressions for Predicting Gestational Diabetes Using Routine Clinical Data: A Retrospective Cohort Study	Ye et al. (2020) [51]	2020	Ob	22,242	104	RC	Dg	To use machine learning methods to predict GDM (Gestational Diabetes) and compare their performance with that of logistic regressions	De
Mortality prediction enhancement in end-stage renal disease (ESRD): A machine learning approach	Macias et al. (2020) [52]	2020	Ne	261	NA	RC	Pg	To assess the potential of the massive use of variables together with machine learning techniques for the improvement of mortality predictive models in ESRD	De
A recurrent neural network approach to predicting haemoglobin trajectories in patients with End-Stage Renal Disease	Lobo et al. (2020) [53]	2020	Ne	1972 patients **	NA	RC	Dg	To develop a RNN approach that uses historical data together with future ESA and iron dosing data to predict the 1-, 2-, and 3-month Hgb levels of patients with ESRD-induced anaemia	Re
Early diagnosis of bloodstream infections in the intensive care unit using machine-learning algorithms	Roimi et al. (2020) [54]	2020	IC	2351 + 1021	NA	RC	Dg	To develop a machine-learning (ML) algorithm that can predict intensive care unit (ICU)-acquired bloodstream infections (BSI) among patients suspected of infection in the ICU	De
Dynamic readmission prediction using routine postoperative laboratory results after radical cystectomy	Kirk et al. (2020) [55]	2020	Ur	996	15	RC	Pg	To determine if the addition of electronic health record data enables better risk stratification and 30-day readmission prediction after radical cystectomy	De
A Machine Learning–Based Model to Predict Acute Traumatic Coagulopathy (ATC) in Trauma Patients Upon Emergency Hospitalization	Li et al. (2020) [56]	2020	EM	818 retrospective, 578 prospective	6 **	PC	Dg	To develop and validate a prediction model for ATC that is based on objective indicators which are already routinely obtained as patients are admitted at the hospital	De
Improved prediction of dengue outbreak using combinatorial feature selector and classifier based on entropy weighted score based optimal ranking	Balamurugan et al. (2020) [57]	2020	ID	480	20 **	RC	Dg	To analyse the performance of the proposed EWSORA Feature Selector, detailed experimentation is conducted on various ML classifiers	De
Using a machine learning approach to predict mortality in critically ill influenza patients: a cross-sectional retrospective multicentre study in Taiwan	Hu et al. (2020) [58]	2020	IC	336	76	RC	Pg	To establish an explainable ML model for predicting mortality in critically ill influenza patients using a real-world severe influenza data set (first 7 days)	De
A novel and simple machine learning algorithm for preoperative diagnosis of acute appendicitis in children	Aydin et al. (2020) [59]	2020	PS	7244	NA	RC	Dg	Provide an easily interpretable model to understand the relationship between blood variables and appendicitis to create an automated decision support tool in the future	De
Identification of risk factors for patients with diabetes: diabetic polyneuropathy case study	Metsker et al. (2020) [60]	2020	En	5425	29–31	RC	Pg	Early identification of the risk of diabetes polyneuropathy based on structured electronic medical records	De
Feasibility of machine learning based predictive modelling of postoperative hyponatremia after pituitary surgery	Voglis et al. (2020) [61]	2020	Ns	207	26	RC	Pg	Evaluate the feasibility of predictive modelling of postoperative hyponatremia after pituitary tumour surgery using preoperative available variables	Re

* It was chosen as the most useful, although it was not the best performer; ** Different models were trained with a different number of features; *** A comparison of the ML models was not made; NA: Not available; NE: Not evaluable (meaning not pertinent). For all the other abbreviations, see Appendix B.

**Table 2:** The 44 reviewed articles reporting validation, which machine learning (ML) models were compared, the best performing model, the metric used by the authors to evaluate the models, results of the studies, most relevant laboratory features and issues of the studies.

Reference	Validation	Comparison	Best Performer	BP’s family	Metrics Used	Results		Most Important Laboratory Features for the Model	Issues/Notes
Awad et al. (2017) [18]	CV	RF, DT, NB, PART, Scores (SOFA, SAPS-I, APACHE-II, NEWS, qSOFA)	RF	Trees	AUROC	RF best performance (VS subset) predicting hospital mortality: 0.90 ± 0.01 AUROC AUROC RF (15 variables) at 6 h: 0.82 ± 0.04 SAPS at 24 h (best performer among scores): 0.650 ± 0.012		Vital Signs, age, serum urea nitrogen, respiratory rate max, heart rate max, heart rate min, creatinine max, care unit name, potassium min, GCS min and systolic blood pressure min	Performance metrics for comparison referred to cross-validation results
Escobar et al. (2017) [19]	CV	3 LoR models, Zilberberg model	LoR (automated model)	Regression	AUROC, pseudo-R2, Sensitivity, Specificity, PPV, NPV, NNE, NRI, IDI		AUROC; R2		Performance metrics for comparison referred to cross-validation results
						Age ≥ 65 years	0.546; −0.1131		
						Basic model	0.591; −0.0910		
						Zilberberg model	0.591; −0.0875		
						Enhanced model	0.587; −0.0924		
						Automated model	0.605; −0.1033		
Richardson and Lidbury (2017) [20]	CV	RF (variables selection) + SVM ***	NE	SVM ***	AUROC, F1, Sensibility, Specificity, Precision	For both HBV and HCV, 3 balancing methods and 2 feature selectors were tested, showing how they can change SVM performances		HBV: ALT, Age and Sodium HCV: Age, ALT and Urea	
Zhang et al. (2017) [21]	CV	GBT ***	NE	Ensemble ***	RI, H-statistic (features) AUROC, Sensibility, Specificity (model)	WBC count ≥ 15 × 109/L (RI: 49.47, *p* < 0.001), spinal cord involvement (RI: 26.62, *p* < 0.001), spinal nerve roots involvement (RI: 10.34, *p* < 0.001), hyperglycaemia (RI: 3.40, *p* < 0.001), brain or spinal meninges involvement (RI: 2.45, *p* = 0.003), EV-A71 infection (RI: 2.24, *p* < 0.001). Interaction between elevated WBC count and hyperglycaemia (H statistic: 0.231, 95% CI: 0–0.262, *p* = 0.031), between spinal cord involvement and duration of fever (H statistic: 0.291, 95% CI: 0.035–0.326, *p* = 0.035), and between brainstem involvement and body temperature (H statistic: 0.313, 95% CI: 0–0.273, *p* = 0.017) GBT model: 92.3% prediction accuracy, AUROC 0.985, Sensibility 0.85, Specificity 0.97			
Takeuchi et al. (2017) [22]	OOB	Scores (Gunma Score, Kurume Score and Osaka Score), RF	RF	Trees	AUROC, Sensibility, Specificity, PPV, NPV, Out OF Bag error estimation	RF: AUROC 0.916, Sensitivity 79.7%, Specificity 87.3%, PPV 85.2%, NPV82.1%, OOB error rate 15.5% Sensitivity and Specificity were: 69.8% and 60.0% GS; 60.6% and 55.4% KS; 24.1% and 77.0% OS. PPV (28.2%–45.1%), NPV (82.0%–86.8%)		Aspartate aminotransferase, lactate dehydrogenase concentrations, percent neutrophils	Performance metrics for comparison referred to cross-validation results
Hernandez et al. (2017) [23]	CV	DT, RF, SVM, Naive Bayes	SVM	SVM	AUROC, AUPRC, Sensitivity, Specificity, PPV, NPV, TP, FP, TN, FN	SVM with SMOTE sampling method and considering 6 features obtained the best results AUROC, AUCPR, Sensibility, Specificity 0.830, 0.884, 0.747, 0.912			
Bertsimas et al. (2018) [24]	VS	LoR, Regularized LoR, Optimal Classification Tree, CART, GBT	Optimal Classification Tree *	Trees	Accuracy (threshold 50%), PPV at Sensibility of 0.6, AUC	Optimal Classification Tree results 60-day mortality, 90-day, 120-day Accuracy: 94.9, 93.3, 86.1 PPV: 20.2, 27.5, 43.1 AUC: 0.86, 0.84, 0.83		Albumin, change in weight, Pulse, WBC count, Haematocrit according to the kind of cancer	The validation set was used only for NN, KNN, and SVM
Jeong et al. (2018) [25]	CV	CERT, CLEAR, PACE, RF, L1-regularized LoR, SVM, NN	RF	Trees	AUROC, F1, Sensibility, Specificity, PPV, NPV	ML models produced higher averaged F1-measures (0.629–0.709) and AUROC (0.737–0.816) compared to those of the original methods AUROC (0.020–0.597) and F1 (0.475–0.563)			
Rosenbaum and Baron (2018) [26]	NA	Univariate models, LoR, SVM	SVM	SVM	AUC, Specificity, PPV	AUROC on testing set (simulated WIBT) best univariate (BUN): 0.84 (interquartile range 0.83–0.84) SVM (difference and values): 0.97 (0.96–0.97) LoR (Difference and values): 0.93		Difference and Values together	Not available data from the comparison among machines
Ge et al. (2018) [27]	CV	RNN-LSTM + LoR vs LoR	RNN-LSTM	DL	AUROC, TP, FP	AUROC cross-validation, AUROC testing set Logistic Regression: 0.7751, 0.7412 RNN-LSTM model: 0.8076, 0.7614		Associated with ICU Mortality: Do Not Reanimate, Prednisolone, Disseminated intravascular coagulation; Associated with ICU Survival: Arterial blood gas pH, Oxygen saturation, Pulse	
Jonas et al. (2018) [28]	CV	LoR (LASSO), RF ***	NE	NE	NE	LASSO identified as the most predictive of a positive response to vasoreactivity test: 6-MWD, diabetes, HDL-C, creatinine, right atrial pressure, and cardiac index RF identified as the most predictive: NT-proBNP, HDL-C, creatinine, right atrial pressure, and cardiac index 6-MWD, HDL-C, hs-CRP, and creatinine levels best discriminated between long-term-responder and not			Performance metrics for comparison referred to cross-validation results Tool available online
Sahni et al. (2018) [29]	NA	LoR, RF	RF	Trees	AUROC	AUROC RF (demographics, physiological, lab, all comorbidities) 0.85 (0.84–0.86) LoR (demographics, physiological, lab, all comorbidities) 0.91 (0.90–0.92)		Age, BUN, platelet count, haemoglobin, creatinine, systolic blood pressure, BMI, and pulse oximetry readings	Performance metrics for comparison referred to cross-validation results
Rahimian et al. (2018) [30]	CV	CPH, RF, GBC	GBC	Ensemble	AUROC	AUROC (CI95), internal validation variables, CPH, RF, GBC QA: 0.740 (0.739, 0.741), 0.752 (0.751, 0.753), 0.779 (0.777, 0.781) T: 0.805 (0.804, 0.806), 0.825 (0.824, 0.826), 0.848 (0.847, 0.849) external validation QA: 0.736, 0.736, 0.796 T: 0.788, 0.810, 0.826		age, cholesterol ratio, haemoglobin, and platelets, frequency of lab tests, systolic blood pressure, number of admissions during the last year	Tool available online
Foysal et al. (2019) [31]	CV	Regression analysis and SVM ***	NE	SVM	R2 score, Standard error of detection, Accuracy	Accuracy: 98%		NE	Performance metrics for comparison referred to cross-validation results
Xu et al. (2019) [32]	CV	L1 Logistic Regression, Regress and Round, Naive Bayes, NN-MLP, DT, RF, AdaBoost, XGBoost.	XGBoost, RF	NA	AUROC, Sensitivity, Specificity, NPV, PPV	Mean AUROC: 0.77 on testing set AUROC > 0.90 on 22 lab tests out of 43 On external validation: results were different according to lab test considered		NE	DL missed Albumin as OS predictor
Burton et al. (2019) [33]	CV	Heuristic model (LoR) with microscopy thresholds, NN, RF, XGBoost	XGBoost *	Ensemble	AUROC, Accuracy, PPV, NPV, Sensitivity, Specificity, Relative Workload Reduction (%)	AUC Accuracy PPV NPV Sensitivity (%) Specificity (%) Relative Workload Reduction (%) Pregnant patients 0.828, 26.94, 94.6 [±0.56], 26.84 [±1.88], 25.29 [±0.92] Children (<11 years) 0.913, 62.00, 94.8 [±0·88], 55.00 [±2.12], 46.24 [±1.48] Pregnant patients 0.894, 71.65, 95.3 [±0.24], 60.93 [±0.65], 43.38 [±0.41] Combined performance 0.749, 65.65, 47.64 [±0.51], 97.14 [±0.28], 95.2 [±0.22], 60.93 [±0.60], 41.18 [±0.39]		WBC count, Bacterial count, Age, Epithelial cell count, RBC count	
Fillmore et al. (2019) [34]	CV	L1 LoR (LASSO), SVM, RF	RF	Trees	Accuracy	LabTest: LR, SVM, RF ALP: 0.98, 0.97, 0.98 ALT: 0.98, 0.94, 0.92 ALB: 0.97, 0.92, 0.98 HDLC: 0.98, 0.91, 0.98 Na: 0.97, 0.98, 0.99 Mg: 0.97, 0.95, 0.99 HGB: 0.97, 0.95, 0.99			Not provided precise data of the performances on testing set
Zimmerman et al. (2019) [35]	CV	LiR, LoR, RF, NN-MLP	NN-MLP	DL	AUROC, Accuracy, Sensitivity, Specificity, PPV, NPV	LiR Regression task: RMSEV Linear Backward Selection Model 0.224 Linear All Variables Model 0.224 AUROC, Accuracy, Sensitivity, Specificity, PPV, NPV LR, Backward Selection Model: 0.780, 0.724, 0.697, 0.730, 0.337, 0.924 LR, All Variables Model: 0.783, 0.729, 0.698, 0.736, 0.342, 0.925 RF, Backward Selection Model: 0.772, 0.739, 0.660, 0.754, 0.346, 0.918 RF, All Variables Model: 0.779, 0.742, 0.673, 0.756, 0.352, 0.921 MLP, Backward Selection Model: 0.792, 0.744, 0.684, 0.756, 0.356, 0.924 MLP, All Variables Model: 0.796, 0.743, 0.694, 0.753, 0.357, 0.926		Sex, age, ethnicity, Hypoxemia, mechanical ventilation, Coagulopathy, calcium, potassium, creatinine level	Performance metrics for comparison referred to cross-validation results
Sharafoddini et al. (2019) [36]	CV	LASSO for choosing most important variables. DT, LoR, RF, SAPS-II (score)	Logistic Regression	Regression	AUROC	Including indicators improved the AUROC in all modelling techniques, on average by 0.0511; the maximum improvement was 0.1209		BUN, RDW, anion gap all 3 days. day 1: TBil, phosphate, Ca, and Lac day 2&3: Lac, BE, PO2, and PCO2 day 3: PTT and pH	
Matsuo et al. (2019) [37]	CV	NN, CPH, CoxBoost, CoxLasso, Random Survival Forest	NN	DL	Concordance Index, Mean Absolute Error	Progression-free survival (PFS): Concordance index, Mean absolute error (mean ± standard error) CPH: 0.784 ± 0.069, 316.2 ± 128.3 DL: 0.795 ± 0.066, 29.3 ± 3.4 Overall survival (OS): CPH: 0.607 ± 0.039, 43.6 ± 4.3 DL: 0.616 ± 0.041, 30.7 ± 3.6		PFS: BUN, Creatinine, Albumin, (Only DL) WBC, Platelet, Bicarbonate, Haemoglobin OS: BUN (only DL) Bicarbonate (only CPH) Platelet, Creatinine, Albumin	
Yang et al. (2019) [38]	OOB	RF ***	NE	Trees ***	OOB	Predicting Outcome (discharge/death) Out-of-bag error 0.073 Accuracy: 0.927 Recall/sensitivity: 0.702 Specificity: 0.973 Precision: 0.840		bicarbonate, phosphate, anion gap, white cell count (total), PTT, platelet, total calcium, chloride, glucose and INR	Not clear how they split dataset and which results are reported
Daunhawer et al. (2019) [39]	CV	L1 Regularized LoR (LASSO), RF	RF+LASSO	NE	AUROC	AUROC cross-validation test set external set RF: 0.933 ± 0.019, 0.927, 0.9329 LASSO: 0.947 ± 0.015, 0.939, 0.9470 RF + LASSO: 0.952 ± 0.013, 0.939, 0.9520		Gestational Age, weight, bilirubin level, and hours since birth	
Estiri et al. (2019) [40]	Pl	CAD (Standard deviation and Mahalanobis distance), Hierarchical k-means	Hierarchical k-means	Clustering	FP, TP, FN, TN, Sensitivity, Specificity, and fallout across the eight thresholds	Specificity increases as threshold decreases. The lowest was 0.9938 Sensitivity in 39/41 variable > 0.85, Troponin I = 0.0545, LDL = 0.4867 About sensitivity, 39/41 CAD~ML, 9/41 CAD > ML About FP, in 45/50 ML had less FP than CAD			
Kayhanian et al. (2019) [41]	CV	LoR, SVM	SVM	SVM	Sensitivity, Specificity, AUC, J-statistic	Sensitivity, Specificity, J-statistic, AUC Linear model, all variables: 0.75, 0.99, 0.7, 0.9 Linear model, three variables: 0.71, 0.99, 0.74, 0.83 SVM, all variables: 0.63, 1, 0.79, N/A SVM, three variables: 0.8, 0.99, 0.63, N/A		Lactate, pH and glucose	
Wang et al. (2019) [42]	CV	Auto-Weka (39 ML algorithms)	RF	Trees	Sensitivity, Specificity, AUROC, Accuracy	Time after ICH, Case number, Best algorithms Sensitivity, Specificity, Accuracy, AUC 1-month: 307 Random forest, 0.774, 0.869, 0.831, 0.899 6 months: 243 Random forest, 0.725, 0.906, 0.839, 0.917		1 month: ventricle compression, GCS, ICH volume, location, Hgb; 6 months: GCS, location, age, ICH volume, gender, DBP, WBC	Connection between HDL-C and reactivity of the pulmonary vasculature is a novel finding
Ye et al. (2019) [43]	NA	Retrospective: RF, XGBoost, Boosting, SVM, LASSO, KNN Prospective: RF	RF	Trees	AUROC, PPV, Sensitivity, Specificity	RF’s AUROC: 0.884 (highest among all other ML models) high-risk sensitivity, PPV, low–moderate risk sensitivity, PPV EWS: 26.7%, 69%, 59.2%, 35.4% ViEWS: 13.7%, 35%, 35.7%, 21.4%		Diagnoses of cardiovascular diseases, congestive heart failure, or renal diseases	No information about tuning
Yang et al. (2020) [44]	CV	LoR, DT (CART), RF, and GBDT	GBDT	Ensemble	AUROC, sensitivity, specificity, agreement with RT-PCR (Agr-PCR)	AUROC; Sensitivity; Specificity; Agr-PCR GBDT 0.854 (0.829–0.878); 0.761 (0.744–0.778); 0.808 (0.795–0.821); 0.791 (0.776–0.805); on cross-validation; GBDT 0.838; 0.758; 0.740 on independent testing set		LDH, CRP, Ferritin	No information about model, training, validation, test
Ma et al. (2020) [45]	CV	RF, XGBoost, LoR for selecting variables for the new model New Model vs Score (CURB-65), XGBoost	New Model	Other	AUROC	AUROC on testing set (13 patients), AUROC on cross-validation New Model: 0.9667, 0.9514 CURB-65: 0.5500, 0.8501 XGBoost: 0.3333, 0.4530		LDH, CRP, Age	Tool available online
Hyun et al. (2020) [46]	NE	k-means***	NE	Clustering***	NE	3 Clusters Cluster 2: abnormal haemoglobin and RBC Cluster 3: highest mortality, intubation, cardiac medications and blood administration		BUN, creatinine, potassium, haemoglobin, and red blood cell	
Lee et al. (2020) [47]	CV	RF, SVM, LASSO, Ridge, Elastic Net Regulation, MEWS	RF	Trees	AUROC, AUPRC, BA, Sensitivity, Specificity, F1, PLR, and NLR	AUROC AUPRC Sensitivity Specificity RF OSO: 0.80 (0.76 to 0.84); 0.25 (0.18 to 0.33); 0.70 (0.62 to 0.82); 0.78 (0.66 to 0.83) RF OSR: 0.88 (0.85 to 0.91); 0.39 (0.30 to 0.47); 0.81 (0.76 to 0.89); 0.81 (0.75 to 0.83)		OSO: Troponin I, creatine kinase and CK-MB; OSR: Lactic Acid	Performance metrics for comparison referred to cross-validation results
Morid et al. (2020) [48]	CV	RF, XGBT, Kernel-based Bayesian Network, SVM, LoR, Naive Bayes, KNN, ANN	RF	Trees	AUC, F1, Accuracy	RF Model performances according to the detection method, Accuracy AUC Last recorded Value: 0.581, 0.589 Symbolic pattern detection: 0.706, 0.694 Local structural pattern: 0.781, 0.772 Global structural pattern: 0.744, 0.730 Local & Global: 0.813, 0.809		NE	
Yu et al. (2020) [49]	NA	ANN***	NE	DL ***	Checking Proportions (CP), Prediction Accuracy, Aggregated Accuracy (AA)	Threshold for CP.AA. performing test 0.15: 90.14%; 95.83% 0.25: 85.78%; 95.05% 0.35: 79.71%; 93.32% 0.45: 71.70%; 90.95% 0.6: 50.46%; 85.30%		NE	Not included data about performances, but only graph of AUROC of prediction to 1 month (with 4-month history)
Chicco and Jurman (2020) [50]	VS	LiR, RF, One-Rule, DT, ANN, SVM, KNN, Naive Bayes, XGBoost	RF	Trees	MCC, F1, Accuracy, TP, TN, PRAUC, AUROC	MCC F1 Accuracy TP TN PRAUC AUROC All features RF + 0.384, 0.547, 0.740, 0.491, 0.864, 0.657, 0.800 Cr+ EF RF +0.418 0.754 0.585 0.541 0.855 0.541 0.698 Cr+EF+FU time LoR +0.616 0.719 0.838 0.785 0.860 0.617 0.822		Serum Creatinine and Ejection Fraction	
Ye et al. (2020) [51]	CV	GDBT, AdaBoost, LGB, Logistic, Vote, XGB, Decision Tree, and Random Forest, stepwise LoR, LoR with RCS	GDBT	Ensemble	AUROC, Recall, Precision, F1	Discrimination AUC GDBT 73.51%, 95% CI 71.36%–75.65% LoR with RCS 70.9%, 95% CI 68.68%–73.12% 0.3 and 0.7 were set as cut-off points for predicting outcomes (GDM or adverse pregnancy outcomes)		GBDT: Fasting blood glucose, HbA1c, triglycerides, and maternal BMI LoR: HbA1c and high-density lipoprotein	
Macias et al. (2020) [52]	CV	RF (features) + RNN-LSTM, RF	RNN-LSTM (all variables)	DL	AUROC	AUROC mortality prediction 1 month RF 0.737 RNN (many) expert variables 0.781 ± 0.021 RNN RF variables 0.820 ± 0.015 RNN all variables 0.873 ± 0.021			
Lobo et al. (2020) [53]	VS	RNN-LSTM + NN + RNN-LSTM ***	NE	DL	Mean Error (ME), Mean Absolute Error (MAE), Mean Squared Error (MSE)	Best model performance ME: 0.017; MAE: 0.527; MSE: 0.489; predicting to 1 month with 5 month of history data			
Roimi et al. (2020) [54]	CV	6 RF+2 XGBoost, RF, XGBoost, LoR	6 RF+2 XGBoost	Other	AUROC, Brier score	Modelling approach BIDMC RHCC AUROC Derivation set, CV Validation set, Derivation set, CV Validation set Logistic-regression: 0.75 ± 0.06, 0.70 ± 0.02, 0.80 ± 0.08, 0.72 ± 0.02 Random-Forest: 0.82 ± 0.03, 0.85 ± 0.01, 0.90 ± 0.03, 0.88 ± 0.02 Gradient Boosting Trees: 0.84 ± 0.04, 0.84 ± 0.02, 0.93 ± 0.04, 0.88 ± 0.01 Ensemble of models: 0.87 ± 0.03, 0.89 ± 0.01, 0.93 ± 0.03, 0.92 ± 0.01 validating the models of BIDMC over RHCC dataset and vice versa, the AUROCs of the models deteriorated to 0.59 ± 0.07 and 0.60 ± 0.06 for BIDMC and RHCC		Most of the strongest features included patterns of change in the time-series variables	Performance metrics for comparison referred to cross-validation results
Kirk et al. (2020) [55]	NA	SVM (cut-offs features), LoR, Random Forest regression Algorithm	RF	Trees	AUROC	AUROC baseline clinical and demographic values 0.52 inclusion of laboratory value thresholds from the day of discharge 0.54 add daily postoperative laboratory thresholds to the demographic and clinical variables 0.59 add postoperative complications 0.62 random forest regression all features 0.68		white blood cell count, bicarbonate, BUN, and creatinine	
Li et al. (2020) [56]	VS	RF, LoR	LoR	Regression	AUROC, Accuracy, Precision, F1, Recall	Prospective cohort results AU-ROC Accuracy Precision F1 score Recall RF: 0.830 (0.770–0.887), 0.916 (0.891–0.936), 0.907 (0.881–0.928), 0.901 (0.874–0.922), 0.917 (0.892–0.937) LoR: 0.858 (0.808–0.903), 0.905 (0.879–0.926), 0.887 (0.859–0.910), 0.883 (0.855–0.906), 0.905 (0.879–0.926)		RBC, SI, BE, Lac, DBP, pH	
Balamurugan et al. (2020) [57]	CV	Auto-Weka (Naive Bayes, DT-J48, MLP, SVM) & 4 features selectors ***	NE	NE	AUROC, F1, Precision, Accuracy, Recall, MCC, TPR, FPR	Proposed model: features selected; Accuracy; TP Rate; FP Rate GA + J48: 9; 94.32; 0.925; 0.118; PSO + J48: 9; 96.25; 0.963; 0.163; CFS + J48: 11; 84.63; 0.861; 0.871; EWSORA + J48; 4; 98.72; 0.950; 0.165;		RBC, HGB, HCT, WBC	Performance metrics for comparison referred to cross-validation results
Hu et al. (2020) [58]	CV	XGBoost, RF, LR, Score (APACHE II, PSI)	XGBoost	Ensemble	AUROC	AUROC XGBoost 0.842 (95% CI 0.749–0.928) RF 0.809 (95% CI 0.629–0.891) LR 0.701 (95% CI 0.573–0.825) APACHE II 0.720 (95% CI 0.653–0.784) PSI 0.720 (95% CI 0.654–0.7897)		Fluid balance domain, Laboratory data domain, severity score domain, Management domain, Demographic and symptom domain, Ventilation domain	
Aydin et al. (2020) [59]	CV	Naïve Bayes, KNN, SVM, GLM, RF, and DT	DT *	Trees	AUC, Accuracy, Sensitivity, Specificity	AUC (%) Accuracy (%) Sensitivity (%) Specificity (%) RF 99.67; 97.45; 97.79; 97.21 KNN 98.68; 95.58; 95.08; 95.93 NB 98.71; 94.76; 94.06; 95.25 DT 93.97; 94.69; 93.55; 96.55 SVM 96.76; 91.24; 90.32; 91.86 GLM 96.83; 90.96; 90.66; 91.16		Platelet distribution width (PDW), white blood cell count (WBC), neutrophils, lymphocytes	
Metsker et al. (2020) [60]	CV	KNN for clustering data and then comparison among Linear Regression, Logistic Regression, ANN, DT, and SVM	ANN	DL	AUROC, F1, Precision, Accuracy, Recall	Model Precision Recall F1 score Accuracy AUC 29’s variables Linear Regression 0.6777, 0.7911, 0.7299 0.7472 31’s variables ANN 0.7982, 0.8152, 0.8064, 0.8261, 0.8988		Age, Mean Platelet Volume	
Voglis et al. (2020) [61]	Bt	Generalized Linear Models (GLM), GLMBoost, Naïve Bayes classifier, and Random Forest	GLMBoost	Ensemble	AUROC, Accuracy, F1, PPV, NPV, Sensibility, Specificity	AUROC: 84.3% (95% CI 67.0–96.4) Accuracy: 78.4% (95% CI 66.7–88.2) Sensitivity: 81.4% Specificity: 77.5% F1 score: 62.1% NPV (93.9%) PPV (50%)		preoperative serum prolactin preoperative serum insulin-like growth factor 1 level (IGF-1) BMI preoperative serum sodium level	

* It was chosen as the most useful, although it was not the best performer; ** Different models were trained with a different number of features; *** A comparison of the ML models was not made; NA: Not available; NE: Not evaluable (meaning not pertinent). For all the other abbreviations, see Appendix B.

**Table 3:** Analysed articles based on year of publication and medical specialty.

Specialty	2017	2018	2019	2020
Cardiology	0	1	0	1
Emergency Medicine	0	0	0	2
Endocrinology	0	0	0	1
Intensive Care	1	1	3	4
Infectious Disease	2	1	0	3
Internal Medicine	0	1	1	0
Laboratory Medicine	1	1	5	1
Nephrology	0	0	0	2
Neurosurgery	0	0	2	1
Obstetrics	0	0	0	1
Oncology	0	1	1	0
Paediatric Surgery	0	0	0	1
Paediatrics	2	0	1	0
Pharmacology	0	1	0	0
Urology	0	0	0	1
Total	6	7	13	18

## Data Availability

Data sharing not applicable

## Appendix A

Scopus query: TITLE-ABS-KEY ([“machine learning”] OR [“deep learning”]) AND TITLE-ABS-KEY ([“laboratory test”] OR [“laboratory medicine”]) AND SUBJAREA (medi) AND DOCTYPE (ar OR re) AND PUBYEAR > 2016.

PubMed query: (“machine learning” [Title/Abstract] OR “deep learning” [Title/Abstract]) AND (“laboratory medicine”[Title/Abstract] OR “laboratory test” [Title/Abstract]).

## Appendix B

Abbreviations used in Table 1 and Table 2.

6MWD	6-minutewalkingdistance
AA	AggregatedAccuracy
AdaBoost	AdaptiveBoosting
ADR	AdverseDrugReactions
AG	AnionGap
ANN	ArtificialNeuralNetwork
APACHE	AcutePhysiologyandChronicHealthEvaluation
ATC	AcuteTraumaticCoagulopathy
AUC	AreaUndertheCurve
AUPRCorPRAUC	AreaUnderthePrecisionRecallCurve
AUROC	AreaUndertheCurveofReceiverOperatingCharacteristic
BA	BalancedAccuracy
BE	BaseExcess
BIDMC	BethIsraelDeaconessMedicalCenter
BSI	bloodstreaminfection
Bt	Bootstrapping
Ca	Cardiology
CART	ClassificationandRegressionTree
CERT	ComparisonofExtremeLaboratoryTest
CFS	CorrelationbasedFeatureSelection
Ch	Characterization
Cl	Clusterisation
CLEAR	ComparisonofExtremeAbnormalityRatio
CP	CheckingProportion
CPH	CoxProportionalHazard
Cr	Creatinine
CURB-65	confusion,urea,respiratoryrate,bloodpressure,>65years
CV	Cross-validation
DBP	DiastolicBloodPressure
De	Detection
Dg	Diagnosis
DL	DeepLearning
DT	DecisionTree
DT-J48	DecisionTreeJ48
EF	EjectionFraction
EHR	ElectronicHealthRecords
EM	EmergencyMedicine
EMPICU	EarlyMortalityPredictionforIntensiveCareUnitpatients
En	Endocrinology
ESA	erythropoiesisstimulatingagents
ESRD	End-StageRenalDisease
EWS	earlywarningsystem
EWSORA	EntropyWeightedScore-basedOptimalRankingAlgorithm
F1	Fscore
FN	FalseNegative
FNR	FalseNegativeRate
FP	FalsePositive
FPR	FalsePositiveRate
FU	Followup
GA	GeneticAlgorithm
GBC	gradientboostingclassifier
GBDTorGDBT	GradientBoostingDecisionTree
GBT	GradientBoostedTree
GCS	GlasgowComaScale
GLM	GeneralizedLinearModels
GS	GunmaScore
HbA1c	GlycatedHaemoglobin
HFMD	Hand-Foot-MouthDisease
IC	IntensiveCare
ICH	intracerebralhaemorrhage
ICU	IntensiveCareUnit
ID	InfectiousDisease
IDI	IntegratedDiscriminationImprovement
IM	InternalMedicine
KD	Kawasakidisease
KNN	K-nearestneighbours
KS	KurumeScore
LASSO	LogisticRegressionwithleastabsoluteshrinkageandselectionoperator
LFA	LateralFlowAssay
LGB	LightGradientBoosting
LiR	LinearRegression
LM	LaboratoryMedicine
LoR	LogisticRegression
LSTM	LongShort-TermMemory
MCC	MatthewsCorrelationCoefficient
MEWS	ModifiedEarlyWarningScore
ML	machinelearning
MLP	Multi-LayerPerceptron
NA	NotAvailable
NB	NaïveBayes
Ne	Nephrology
NE	NotEvaluable
NEWS	NationalEarlyWarningScore
NLR	NegativeLikelihoodRatio
NN	NeuralNetwork
NNE	numberofincidentcasesonewouldneedtoevaluatetodetectonerecurrence
NN-MLP	NeuralNetworkMultilayerPerceptrons
NPV	negativepredictivevalue
NRI	netreclassificationimprovement
Ns	Neurosurgery
NT-proBNP	N-terminalpro–brain-typenatriureticpeptide
Ob	Obstetrics
On	Oncology
OOB	Out-of-Bagerrorestimation
OS	OsakaScore
OSO	orderstatusonly
OSR	orderstatusandresults
PACE	PrescriptionpatternAroundClinicalEvent
PART	ProjectiveAdaptiveResonanceTheory
PC	Prospectivecohort
Pd	Paediatry
PFS	Progression-freesurvival
Pg	Prognosis
Ph	Pharmacology
PLR	PositiveLikelihoodRatio
PPV	PositivePredictiveValue
PS	PaediatricSurgery
PSI	PneumoniaSeverityIndex
PSO	ParticleSwarmOptimization
qSOFA	quickSepsis-relatedOrganFailureAssessment
R2	Nagelkerke’spseudo-R2
RBC	RedBloodCell
RC	RetrospectiveCohort
Rch	Research
Re	Regression
RF	RandomForest
RHCC	RambamHealthCareCampus
RI	relativeimportance
RMSEV	RootMeanSquareErrorValues
RNN	RecurrentNeuralNetwork
RNN-LSTM	RecurrentNeuralNetwork-LongShort-TermMemory
RT-PCR(Agr-PCR)	Real-timereversetranscriptionpolymerasechainreaction
SAPS	SimplifiedAcutePhysiologyScore
SAPS-II	SimplifiedAcutePhysiologyScoreII
SI	ShockIndex
SMOTE	SyntheticMinorityOversamplingTechnique
SOFA	SequentialOrganFailureAssessment
SVM	SupportVectorMachine
TBil	TotalBilirubin
Th	Therapy
TN	TrueNegative
TNR	TrueNegativeRate
TP	TruePositive
TPR	TruePositiveRate
Ur	Urology
ViEWS	VitalPACEarlyWarningScore
VS	ValidationSet
WBC	WhiteBloodCell
WBIT	WrongBloodinTube
XGBorXGBoost	eXtremeGradientBoosting
XGBT	eXtremeGradientBoostingTrees
